# National reporting of deaths after enhanced Ebola surveillance in Sierra Leone

**DOI:** 10.1371/journal.pntd.0008624

**Published:** 2020-08-18

**Authors:** Mohamed F. Jalloh, Reinhard Kaiser, Mariam Diop, Amara Jambai, John T. Redd, Rebecca E. Bunnell, Evelyn Castle, Charles Alpren, Sara Hersey, Anna Mia Ekström, Helena Nordenstedt

**Affiliations:** 1 Division of Global Health Protection, Center for Global Health, Centers for Disease Control and Prevention, Atlanta, Georgia, United States of America; 2 Department of Global Public Health, Karolinska Institutet, Stockholm, Sweden; 3 eHealth Africa, Freetown, Sierra Leone; 4 Ministry of Health and Sanitation, Freetown, Sierra Leone; 5 Karolinska University Hospital Huddinge, Stockholm, Sweden; NIAID Integrated Research Facility, UNITED STATES

## Abstract

**Background:**

Sierra Leone experienced the largest documented epidemic of Ebola Virus Disease in 2014–2015. The government implemented a national tollfree telephone line (1-1-7) for public reporting of illness and deaths to improve the detection of Ebola cases. Reporting of deaths declined substantially after the epidemic ended. To inform routine mortality surveillance, we aimed to describe the trends in deaths reported to the 1-1-7 system and to quantify people’s motivations to continue reporting deaths after the epidemic.

**Methods:**

First, we described the monthly trends in the number of deaths reported to the 1-1-7 system between September 2014 and September 2019. Second, we conducted a telephone survey in April 2017 with a national sample of individuals who reported a death to the 1-1-7 system between December 2016 and April 2017. We described the reported deaths and used ordered logistic regression modeling to examine the potential drivers of reporting motivations.

**Findings:**

Analysis of the number of deaths reported to the 1-1-7 system showed that 12% of the expected deaths were captured in 2017 compared to approximately 34% in 2016 and over 100% in 2015. We interviewed 1,291 death reporters in the survey. Family members reported 56% of the deaths. Nearly every respondent (94%) expressed that they wanted the 1-1-7 system to continue. The most common motivation to report was to obey the government’s mandate (82%). Respondents felt more motivated to report if the decedent exhibited Ebola-like symptoms (adjusted odds ratio 2.3; 95% confidence interval 1.8–2.9).

**Conclusions:**

Motivation to report deaths that resembled Ebola in the post-outbreak setting may have been influenced by knowledge and experiences from the prolonged epidemic. Transitioning the system to a routine mortality surveillance tool may require a robust social mobilization component to match the high reporting levels during the epidemic, which exceeded more than 100% of expected deaths in 2015.

## Author summary

By November 2015 when the World Health Organization declared the Ebola epidemic in Sierra Leone to be over, approximately 95% of the population had become aware of the risk of Ebola transmission linked to physical contact with infected corpses, especially during traditional burials. Enhanced Ebola surveillance was implemented between November 2015 and June 2016, i.e. after the epidemic had officially ended to improve detection of possible new cases. Reporting to the 1-1-7 system declined nationally after enhanced Ebola surveillance ended even though the Government of Sierra Leone continued to mandate that all deaths must be reported. Based on a request from the Sierra Leone Ministry of Health and Sanitation, we conducted a telephone survey with a national sample of people who had reported a death in 2017 after the end of enhanced surveillance to understand their motivations for reporting and describe the deaths that they reported. In addition, we analyzed the five-year trends (2014–2019) in the number of deaths reported through the system. Analysis of monthly summary data of deaths reported showed that on the last month of enhanced surveillance, 3,851 deaths were reported compared to 2,456 deaths in the month immediately after (July 2016). The monthly numbers of reported deaths continued to plummet and reached as low as 1,550 in January 2017, 673 in January 2018, and 586 in January 2019. In the survey, we uncovered that people who reported deaths were mainly motivated to do so in order to comply with the Government’s mandate. After adjusting for potential confounders, motivations to report were strongly associated with the presence of Ebola-like symptoms in the decedent. Additional investigations are needed to unveil reporting barriers among people who failed to report household deaths to the 1-1-7 system to optimize reporting levels. It has been shown that during the Ebola epidemic that it is possible to reach high levels of death reporting in Sierra Leone as exemplified by the fact that in 2015 more than 100% of the expected deaths nationally were reported; albeit not counting potential duplicates. The post-Ebola-outbreak setting provides a unique opportunity to improve future overall mortality surveillance in Sierra Leone and contribute to the establishment of civil registration of vital statistics.

## Introduction

From May 2014 to November 2015, Sierra Leone experienced the largest epidemic of Ebola to date, which also affected neighboring Liberia and Guinea. The epidemic in Sierra Leone resulted in more than 14,000 cases and nearly 4,000 deaths of Ebola [1]. Traditional practices involving physical contact with corpses and sick people [2] contributed to Ebola transmission [3–5]. It has been estimated that an average of approximately 2.5 new Ebola cases resulted from every unsafe traditional burial during the epidemic in West Africa [3]. In one extreme situation, 28 confirmed cases were epidemiologically linked to a single traditional burial of a prominent pharmacist in Moyamba district, Sierra Leone in September 2014 [4]. Out of these 28 cases, approximately 75% of them had direct physical contact with the pharmacists’ corpse. Epidemic control efforts heavily focused on halting risky behaviors, such as washing and touching of corpses as part of traditional burial rites, and providing alternatives for safe burials by specially trained teams [6–8]. There was a need to promptly identify all deaths occurring in communities to test them for Ebola and ensure safe burial [9–11]. Community-based reporting of deaths consequently constituted an important component of responding to the epidemic [5, 12].

In August 2014, the Government of Sierra Leone repurposed an existing national, toll-free telephone line (1-1-7 system) for communities to report all deaths and suspected Ebola patients as part of the epidemic response [13]. The 1-1-7 system’s design, implementation and adaptations have been described elsewhere [14]. Although the Government of Sierra Leone required communities to report deaths of all-causes to the 1-1-7 system during the 2014–2015 Ebola epidemic [14], burial teams were not always successful in responding to all death alerts within 24 hours due to the high community demand and call volumes. This resulted in dissatisfaction among communities where families had to wait longer than 24 hours for safe burial services. Furthermore, communities were at times dissatisfied with how corpses were handled by burial teams [15, 16]. As the epidemic progressed, safe alternatives to traditional burials were made available to families such as observing the burial from a safe distance and allowing a religious leader to pray on the corpse. Religious leaders played a key role in advocating for incorporating safe alternatives that show respect for the deceased and their family [17]. Social mobilization efforts were implemented nationwide to promote Ebola protective behaviors including community acceptance of safe burial measures and reporting of deaths to the 1-1-7 line during the epidemic [9, 10, 18, 19]. After the outbreak ended, social mobilization and risk communication interventions that promoted the use of the 1-1-7 line were scaled down.

Analysis of calling trends indicated that the numbers of deaths reported to the 1-1-7 system sharply declined after the epidemic ended even though the official government policy mandated that all deaths occurring in communities must still be reported. All reported deaths that were suspected to be Ebola or otherwise ‘suspicious’ were supposed to be forwarded to district-based surveillance officers by the 1-1-7 call center operators for screening and further investigations depending on the circumstances of the death.

Strategies for continuing death reporting in Sierra Leone or other post-Ebola-outbreak settings are scarce. Factors contributing to the decline in death reporting after the epidemic and enhanced surveillance ended are not well understood, and neither are motivating factors for those who continued to report. Moreover, the potential influence of Ebola experiences on death reporting motivations in post-Ebola-outbreak settings has not been examined. To inform routine mortality surveillance, we aimed to describe deaths reported to the 1-1-7 system, perceptions of the reporting system, and motivations to report the deaths after the epidemic and enhanced Ebola surveillance had ended.

## Methods and materials

Previous analysis of the number of sick people and deaths reported to the 1-1-7 system has been published for the period of September 2014 to December 2016 [14]. To establish more comprehensive trends in death reporting during a five-year period spanning September 2014—September 2019, we obtained monthly unadjusted aggregated data of death alerts placed to the 1-1-7 call center managed by eHealth Africa on behalf of the Sierra Leone Ministry of Health and Sanitation [20]. To inform strategies for improving routine surveillance of deaths, in April 2017, we then conducted a cross-sectional, telephone-based survey with individuals aged 18 years and above who reported a death to the 1-1-7 system after the end of enhanced Ebola surveillance in Sierra Leone. The methods and materials of the survey have been described in this paper in accordance with guidelines for Strengthening the Reporting of Observational Studies in Epidemiology [21].

### Sampling

In April 2017 we obtained a sampling frame of 7,025 callers who reported a death to the 1-1-7 system. Survey respondents were randomly selected from a stratified sampling frame of all callers who reported at least one death between December 2016 and April 2017. Fewer than 5% of the records were duplicates and were removed from the sampling frame. For callers who reported multiple but non-duplicate deaths (< 10% of sample), the most recent death was kept while all others were removed to mitigate recall bias. We then stratified the sampling frame by geographic region of residence (West, North, East, South). In our sample size calculations, we assumed 70% call-success rate, 50% overall-response rate agreeing to consent, and 90% item-response rate. We aimed to obtain a final sample of approximately 1,355 callers, to allow for a 2.5% margin of error for national estimates and 5.0% margin of error for regional estimates of death-reporting motivations. Significance level was set to α = 0.05. This resulted in a random list of 4,300 people to contact by telephone. Trained interviewers made up to three attempts at different times of the day to contact potential participants. A telephone number was marked as unreachable and removed from the telephone database after three unsuccessful attempts.

### Data collection instruments

The questionnaire development was informed by a focus group discussion with a convenience sample of 12 respondents to assess appropriateness of item formats, respondents’ understanding and interpretation of questions, appropriate sequencing to mitigate bias, and categorization of expected responses to open-ended questions (for example, motivations to report a death). The questionnaire was subsequently revised and piloted with a convenience sample of 25 eligible respondents. Participants in the pilot were excluded from the final sample selection to avoid repeat-interview bias.

### Training and data collection

A team of ten interviewers and two supervisors were trained on the proper administration of the survey including informed consent, oral translations of items from English to common local languages (Krio, Mende, Temne, and Fullah), use of the Open Data Kit (ODK) digital data collection tool [22], and interviewing techniques. The interviews were conducted in respondent’s preferred local language. Calls were placed by interviewers using a telephone system setup within the 1-1-7 Call Center in Freetown. On average, interviews lasted approximately 15–20 minutes. The interviews were administered using ODK (www.opendatakit.org) installed on computer tablets pre-programmed with a digital copy of the questionnaire. Supervisors oversaw the data collection process including monitoring phone interviews, verifying data entered in ODK, and reviewing final submission of processed data to a secured, web-based hosting server. Verbal informed consent was obtained from all participants before initiating the telephone interview.

### Explanatory variables

Sociodemographic variables included region of residence, sex, age, education, religion, and occupation. In addition, we collected data on circumstances surrounding the death including the nature of death (accident-related, possible stillbirth, possible maternal death), signs and symptoms, place of death, and treatment seeking history within the month prior to dying. Call history to 1-1-7 during the Ebola epidemic was defined as anyone who responded “yes” to the question “Did you ever call 1-1-7 during the Ebola crisis in Sierra Leone (May 2014 to November 2015)?” It is worth noting that by 2015 the epidemic response capacity had generally improved compared to 2014 when response capacity was severely challenged as new Ebola cases peaked nationwide in November of that year [23]. Past Ebola experience was dichotomized into “yes” and “no” such that anyone who responded by saying “yes” to one or more of the following three questions was categorized as having some past Ebola experience: (a) “Do you personally know anyone who died from Ebola?” (b) “Do you personally know anyone who survived Ebola?” and (c) “Do you personally know anyone who was quarantined due to Ebola?” Item wording and grouping for past Ebola experience was directly informed by a prior assessment in Sierra Leone [24].

### Outcome variable

Motivations to report a death were captured by asking an open-ended question: “What made you call 1-1-7 to report the death?” Without prompting for any specific responses, interviewers recorded the reason(s) for calling provided by respondents into the following six categories: (a) “find out the cause of death;” (b) “protect self or others from possible infection;” (c) “obey Government policy/law;” (d) “obtain burial permit (to allow traditional burial);” (e) “obtain death certificate;” and (f) “other.” Selection of multiple reasons for calling was allowed, and data collectors probed to get an exhaustive list of motivations.

### Data analysis

**Fig 1:**
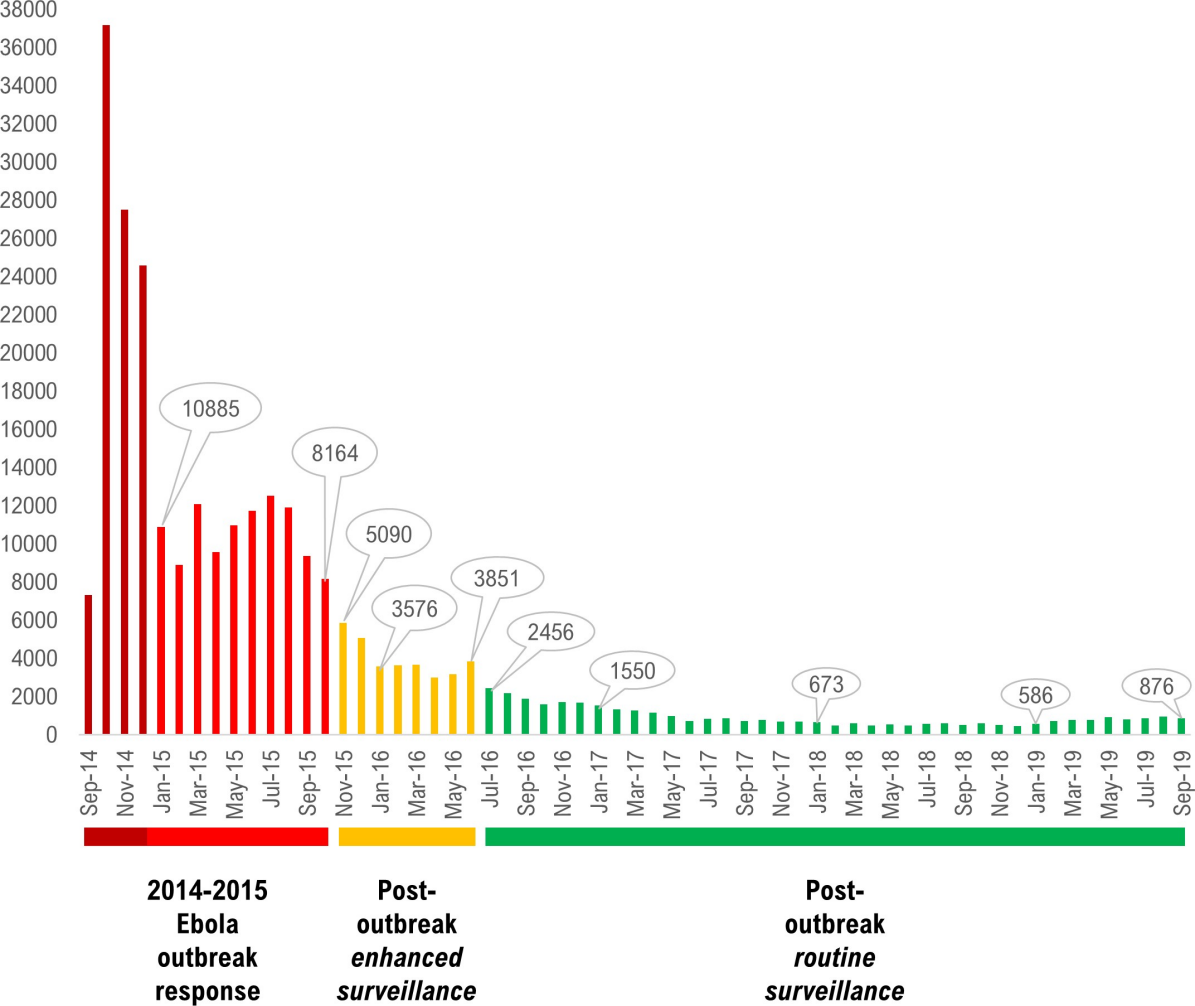
Distribution of the monthly unadjusted aggregated number of deaths reported to the 1-1-7 system, Sierra Leone, 2014–2019.

First, we described trends in reporting by plotting a bar graph (Fig 1) of the raw number of monthly deaths reported to the 1-1-7 line during the (i) Ebola epidemic (September 2014-October 2015), (ii) post-outbreak enhanced surveillance (November 2015-June 2016), and (iii) post-outbreak routine surveillance (July 2016-September 2019). Given the aggregated format of the monthly data of 1-1-7 death alerts, we could not account for potential duplicates in the descriptive analysis.

The survey data were analyzed using Stata version 15 SE (StataCorp LLC, College Station, TX). Frequencies, proportions and other descriptive statistics were generated for all variables. Responses indicating “don’t know”, “don’t remember”, and “declined to respond” were treated as missing values. A composite outcome variable was created for scoring motivations expressed by respondents. The score could range from 0 to 6 depending on the number of motivations that respondents cited. Two composite binary exposure variables were then generated. First, a binary variable was generated to indicate if Ebola-like symptoms (fever, diarrhea, vomiting) were present in the decedent (coded 0 if none and 1 if one or more symptoms). Second, a binary variable was generated for knowing someone who died from Ebola, survived Ebola or quarantined due to Ebola exposure during the 2014–2015 epidemic (coded 0 if none and 1 if one or more such experiences).

Given the ordered outcome for motivations using a count variable, we used ordered logistic regression modeling in our multivariate analyses to estimate odds ratios (ORs) and their 95% confidence intervals (CIs). We fitted a model to examine the possible associations between motivations to report the death and (a) experiencing Ebola-like symptoms before dying, (b) previously calling the 1-1-7 line during the epidemic, and (c) knowing someone who died from Ebola, survived Ebola, or was quarantined due to Ebola during the epidemic in Sierra Leone. The model was adjusted for sociodemographic characteristics of the person who reported the death (region, sex, age, education, religion, health worker status) and of the deceased person (sex, age, religion). Educational attainment and occupation of the deceased persons were excluded in the models due to high frequency of missing values. The covariates in the model were assessed for collinearity. Subsequently, region of residence of the deceased persons was excluded because it was collinear with region of residence of the person who reported the death. In all models, significance level was set to α = 0.05 for a two tailed Wald test.

### Ethical approval

The assessment was approved as non-research by the Sierra Leone Ministry of Health and Sanitation. Participation of U.S. Centers for Disease Control and Prevention (CDC) staff was approved as a non-research activity by CDC’s Center for Global Health (CGH-HSR# 2016–276).

## Results

### Five-year death reporting trends between September 2014 and September 2019

The monthly aggregated data of deaths reported to the 1-1-7 system showed a sharp decline after the 2014–2015 Ebola epidemic ended in Sierra Leone compared to the post-outbreak enhanced mortality surveillance period. For instance, in the final month before the Ebola epidemic was declared over in Sierra Leone (October 2015), a total of 8,164 deaths were reported through the system compared to 5,090 in November 2015 when enhanced surveillance began. Moreover, in the last month of enhanced surveillance (June 2016), 3,851 deaths were reported through the system compared to 2,456 in the beginning of post-outbreak routine surveillance of deaths. The number of deaths reported to the 1-1-7 system continued to plummet to as low as 1,550 in January 2017, 673 in January 2018, and 586 in January 2019. (Fig 1).

In the year when we conducted the survey (2017), a total of 11,642 deaths were reported to the 1-1-7 system compared to 32,469 in 2016 and 117,036 in 2015. Sierra Leone has a crude death rate of 11.9 per 1000 population according to the 2015–2020 estimates by the United Nations [25]. Therefore, approximately 95,000 deaths could be expected yearly in the total estimated population of 8 million. Thus, the number of deaths reported to the 1-1-7 system in 2017 was approximately 12% of expected total deaths in the country compared to approximately 34% in 2016 and over 100% in 2015.

### Description of the respondents

Telephone contact was established with 1,330 individuals out of 4,300 eligible individuals in the sampling frame. Of those who were successfully reached by telephone, 1,291 consented to participate in the survey: 416 (32.2%) from the Northern region, 322 (24.9%) from the Western Region, 280 (21.7%) from the Eastern Region, and 273 (21.1%) from the Southern Region. Most respondents were males (84.8%), and this was consistent across regions. Nearly half (49.5%) of the 196 respondents who identified as female were also health workers. The median age was 40 years (males 39 years and females 37 years). Overall, 10.6% of the respondents had no formal education. About two-thirds (63.8%) of all respondents identified as Muslims and the rest identified as Christians (36.2%). Family members of the deceased reported 55.8% of the deaths. Half of all respondents (52.1%) had previously called the 1-1-7 line at least once during the 2014–2015 Ebola epidemic in Sierra Leone. Of those who called during the epidemic (n = 630), 85.7% reported a death. Two-thirds of all respondents (68.3%) reported past Ebola experiences including knowing someone who died from Ebola (49.2%), survived Ebola (49.9%) or was quarantined due to Ebola exposure (57.7%) (Table 1).

**Table 1:** Descriptions of respondents by sex, Sierra Leone, April 2017.

	All Respondents*		Male		Female	
	N	%	N	%	N	%
**Region**						
West	322	24.9	282	25.8	40	20.4
North	416	32.2	362	33.1	54	27.6
East	280	21.7	231	21.1	49	25.0
South	273	21.1	220	20.1	53	27.0
**Age**						
18–29 years	262	20.3	216	19.8	46	23.5
30–39 years	410	31.8	341	31.1	69	35.2
40–49 years	349	27.0	299	27.3	50	25.5
50 years and above	270	20.9	239	21.8	31	15.8
**Education**						
None	135	10.6	122	11.3	13	6.8
Primary	73	5.7	64	5.9	9	4.7
Secondary and above	1066	83.7	898	82.8	168	88.4
**Religion**						
Muslim	812	63.8	734	67.9	78	40.8
Christian	460	36.2	347	32.1	113	59.2
**Occupation**						
Health worker	347	27.3	253	23.4	94	49.5
Non-health worker	925	72.7	829	76.6	96	50.5
**Relationship to the deceased**						
Family/relative	672	55.8	597	57.9	75	43.1
Friend/neighbor	290	24.1	265	25.7	25	14.4
Other	243	20.1	169	16.4	74	42.4
**Past call to the 1-1-7 system during the epidemic**						
Called at least once in 2014 or 2015	630	52.1	564	54.3	66	38.4
**Past Ebola experiences**						
Knew someone who died	597	49.2	526	50.6	71	41.0
Knew someone who survived	605	49.9	537	51.7	68	39.3
Knew someone was quarantined	699	57.7	612	58.9	87	50.3
Any of the above Ebola experiences	828	68.3	723	69.6	105	60.7

*Total of 1291 respondents (male 1095, female 196). Due to missing values, valid responses were lower for the variables on education (N = 1274; male 1084, female 190), religion (N = 1272; male 1081, female 191), occupation (N = 1272; male 1082, female 190), relationship to the decedent (N = 1205, 1031 male, 174 female), past call to the 1-1-7 system during the epidemic (N = 1210; male 1038, female 172); and past Ebola experiences (N = 1212; male 1039, female 173).

### Description of the deaths reported

In the sample obtained, deceased persons were more frequently male (54.9%), had no education (53.5%), affiliated as Muslim (78.5%), and were 50 years old or above (41.6%) (Table 2).

**Table 2:** Descriptions of the deaths reported in the sample, Sierra Leone, April 2017.

	All deaths*		Male		Female	
	N	%	N	%	N	%
**Region**						
West	324	25.1	174	25.5	150	25.8
North	420	32.5	227	32.0	193	33.2
East	278	21.5	168	23.7	110	18.9
South	269	20.8	140	19.8	129	22.2
**Age**						
<1 year	127	9.8	59	8.3	68	11.7
1–9 years	138	10.7	72	10.2	66	11.3
10–19 years	63	4.9	39	5.5	24	4.1
20–29 years	122	9.5	62	8.7	60	10.3
30–39 years	158	12.2	78	11.0	80	13.8
40–49 years	146	11.3	85	12.0	61	10.5
50 years and above	537	41.6	314	44.3	223	38.3
**Education**						
None	437	53.5	222	49.6	215	58.3
Primary only	102	12.5	45	10.0	57	15.5
Secondary and above	278	34	181	40.4	97	26.3
**Religion**						
Muslim	935	79.6	526	80.8	409	78.1
Christian	240	20.2	125	19.2	115	22.0

*Total of 1291 deaths (male 709, female 582). Due to missing values, valid responses were lower for the variables on education (N = 817; male 448, female 369) and religion (N = 1175; male 651, female 524).

Overall, 376 (29.1%) deaths were women of reproductive age, 127 (9.8%) were infants, and 59 (4.9%) were accident-related deaths. Among deaths of women of reproductive age, 24 (6.4%) were pregnant at the time of the death. Thirty-three of the infant deaths (30.3%) were stillbirths. Overall, 83.3% of deceased persons reportedly received some form of treatment from one or more sources within the past month of dying, and non-exclusively cited the place of treatment as health facility (82.4%), home (18.5%), pharmacy or drug store (10.9%), traditional healer (6.4%), and other sites (<1%). The most frequently cited symptoms that the deceased persons had purportedly experienced within the past month of dying were fever (32.1%), joint pain (21.0%), headache (19.6%), and abdominal pain (15.7%). Ebola-like symptoms (fever, diarrhea, or vomiting) were reportedly experienced by 35.5% of the decedents (Table 3). Missing values were higher for the variables on occupation of the deceased person (n = 474; 36.7%), education level of the deceased person (n = 392; 30.4%), and treatment received before dying (n = 222; 17.2%) when compared to other variables (less than 10%). Missing values were mostly due to reporting of deaths by health workers who did not know certain details about the deceased person.

**Table 3:** Circumstances of the deaths reported in the sample, Sierra Leone, April 2017.

	N*	n	%
**Type of death**			
Women aged 14–49 years	1291	376	29.1
Women aged 14–49 years pregnant at time of death	376	24	6.4
Children under 1 year of age	1291	127	9.8
Children under 1 year of age born dead	109	33	30.3
Accident-related deaths	1195	59	4.9
**Place of death**			
Home / elsewhere in community	1171	747	63.8
Health facility		413	35.3
Other		11	0.9
**Treatment**			
Received any treatment within past month of death	1069	890	83.3
Home	890	165	18.5
Health facility		733	82.4
Pharmacy / drug store		97	10.9
Traditional healer		57	6.4
Other		4	0.4
**Signs and symptoms**			
Fever	1232	396	32.1
Joint pain		259	21.0
Headache		241	19.6
Abdominal pain		193	15.7
Other swelling		92	7.5
Chest pain		90	7.3
Vomiting		57	4.6
Diarrhea		43	3.5
Difficulty breathing		38	3.1
Puffy face		20	1.6
Convulsion		19	1.5
Blurred vision		17	1.4
Vaginal bleeding		13	1.1
**Number of Ebola-like symptoms (fever, diarrhea, vomiting)**			
None	1232	795	64.5
One or more		437	35.5

### Preferences for continuation of the 1-1-7 system

**Fig 2:**
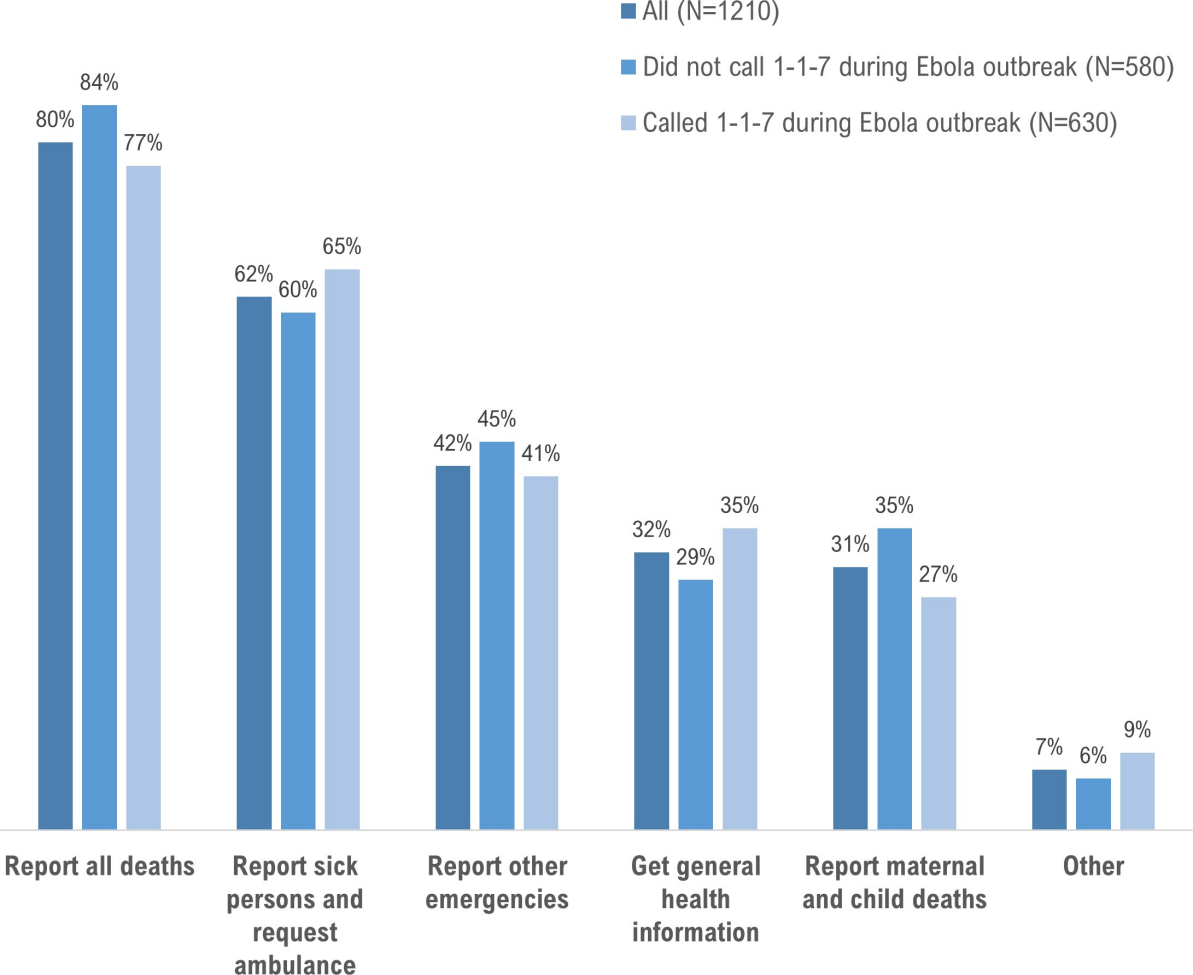
Percent distribution of preferences expressed by respondents for the continuation of the 1-1-7 system, Sierra Leone, 2017. Total of 1174 respondents with complete data.

Nearly all respondents (94.1%) wanted the government to continue using the 1-1-7 system in Sierra Leone, and to keep the current ‘1-1-7’ number (89.7%). Of those who wanted continuation of the reporting system (n = 1174), reporting of all deaths was the most commonly reported preference (79.7%) (Fig 2).

### Motivations to report deaths

**Fig 3:**
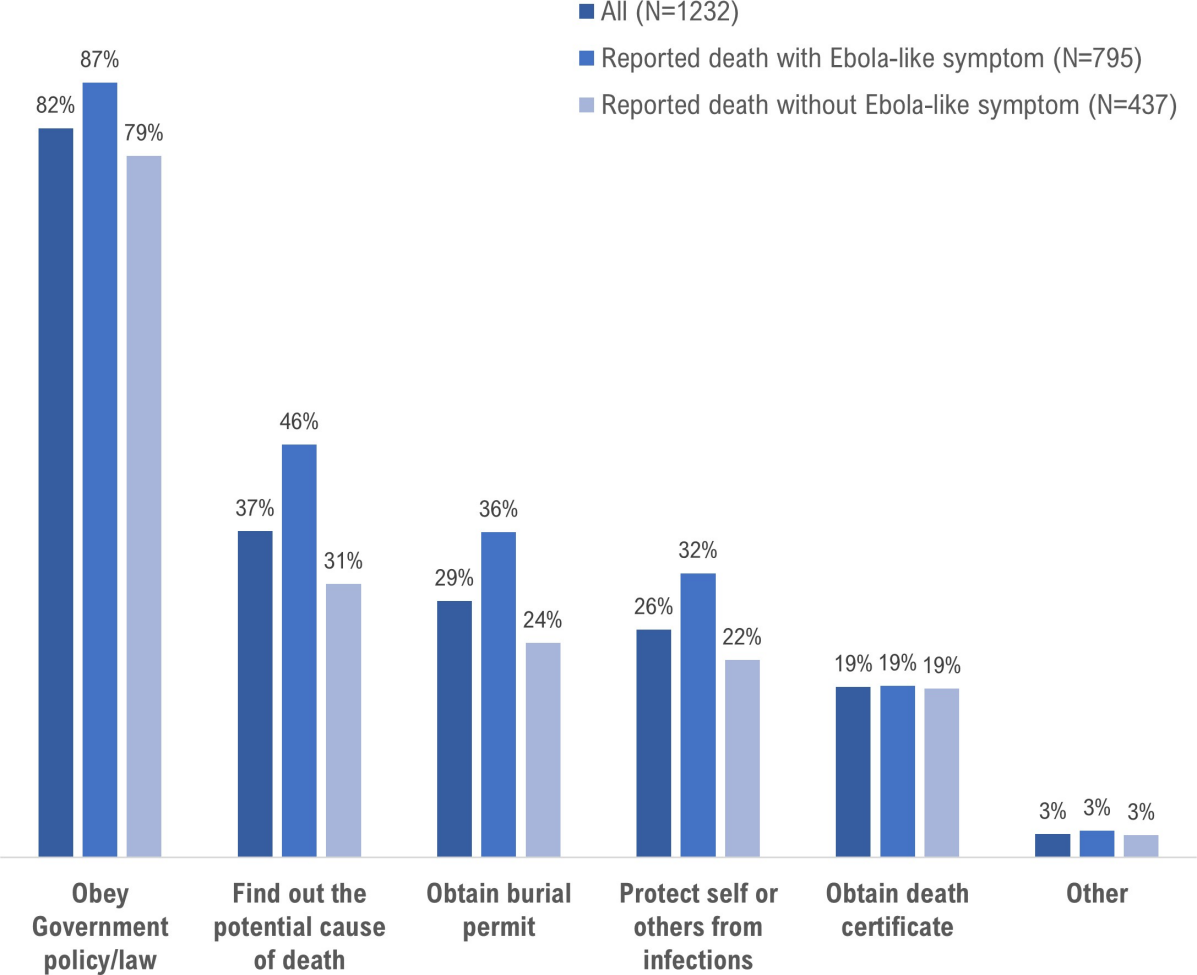
Percent distribution of motivations expressed by respondents for reporting deaths to the 1-1-7 system, Sierra Leone, 2017. Total of 1291 respondents with complete data.

The most frequently cited motivations were to obey government policy (81.6%), find out the cause of death (36.5%), obtain burial permit (28.7%), and protect self or others from infection (25.5%) (Fig 3). Compared to deaths that did not exhibit Ebola-like symptoms, exhibiting one or more Ebola-like symptoms was associated with a two-fold increase in the odds of being motivated to report the death (adjusted OR [aOR] 2.26 CI 1.78–2.87). Motivations to report were not associated with previously calling the 1-1-7 line during the Ebola epidemic (aOR 1.0 CI 0.79–1.27) or knowing someone who died, survived or was quarantined due to Ebola during the epidemic (aOR 0.94 CI 0.73–1.21) (Table 4).

**Table 4:** Past Ebola experiences and Ebola-like symptoms as determinants of death reporting motivations, Sierra Leone, April 2017.

	Motivations to report*	
	aOR (95%CI)	P value †
**Person who died: Experienced symptoms of fever, diarrhea, or vomiting before dying** No Yes	Reference 2.3 (1.8–2.9)	0.000
**Person who reported the death: Previously called 1-1-7 line during the epidemic** No Yes	Reference 1.0 (0.8–1.3)	0.972
**Person who reported the death: Knew someone who died, survived, or was quarantined due to Ebola during the epidemic** No Yes	Reference 0.9 (0.7–1.2)	0.631

*Total of 1,096 respondents included in the model (complete cases); 195 excluded due to missing data for one or more variables.

† Wald statistical p value from ordered logistic regression model. AOR = adjusted for all main exposure variables plus region of residence, sex (of respondent and deceased person), age (of respondent and deceased person), education (of respondent), religion (of respondent and deceased person), occupation (of respondent)

## Discussion

Our descriptive analysis of the five-year trends in the number of deaths reported to the 1-1-7 system identified a substantial decline in reporting after the period of enhanced Ebola surveillance ended. In 2017, the system maximally captured about 12% of the total expected deaths in the country compared to approximately 34% in 2016 and more than 100% (*sic*) in 2015. In the telephone survey we identified motivations related to death reporting that have practical implications for improving routine mortality surveillance in a post-Ebola-outbreak setting. Nearly all respondents wanted the death reporting system to continue. The leading motivation for reporting was the desire to obey the government’s reporting mandate of all deaths. Reasons for this desire to comply with reporting mandate were not directly evaluated in our assessment but may be linked to altruistic intentions to help prevent potential Ebola as documented in a prior qualitative assessment near the end of the epidemic in Sierra Leone [26]. In fact, we found that people who reported deaths that had experienced Ebola-like symptoms were more motivated to report, which may have also been influenced by knowledge and experiences from the prolonged Ebola epidemic.

Effective mortality surveillance is an important pillar of promptly identifying and responding to deaths from notifiable diseases such as Ebola, especially in outbreak-prone areas. Recent epidemics of Ebola in sub-Saharan Africa have reinforced the need for effective surveillance systems including mechanisms to promptly detect cluster-deaths that may be tied to a possible outbreak of Ebola or other infectious diseases [4, 23, 27]. It should be noted that prior to the Ebola outbreak deaths were usually reported in-person to local city councils mainly to obtain a burial plot [14]. Therefore, telephone reporting of deaths to the national government was a new behavior for people in Sierra Leone before the Ebola outbreak.

The context in which a death reporting system is implemented poses ethical considerations. Deaths tended to become widely publicized in communities during the epidemic in Sierra Leone. Anyone in the community could report a death to the 1-1-7 line even without having full information about the details of the death or the approval of close relatives. Given the limited capacity to respond to all incoming death notifications, multiple reports of the same death with incomplete or contradictory information may have made it difficult to prioritize the dispatch of safe burial teams. However, during the post-outbreak period, our survey revealed that mostly family members and health workers reported deaths to the 1-1-7 system, which likely improved the completeness and accuracy of the information provided about the death. Despite confidentiality guidelines, in both the outbreak and post-outbreak contexts it is unclear if callers to the 1-1-7 line were assured confidentiality when they reported a death. In addition, the training guidelines of call operators stated that callers were supposed to be informed that they may receive a follow-up call to get more information about the reported death. We cannot verify whether operators informed all callers about potential follow-up calls. It is possible that concerns about confidentiality may have influenced death reporting behaviors over time, including in our assessment.

Continued implementation of the 1-1-7 death reporting system is just one example of how Sierra Leone leveraged its Ebola response infrastructure for routine surveillance [23]. Another example is seen in how the Government of Sierra Leone, with support from partners, transitioned from a paper-based to a web-based electronic Integrated Disease Surveillance and Response (eIDSR) system after the Ebola epidemic. Sierra Leone became the first country to have a fully functional eIDSR system in sub-Saharan Africa in 2019 [28]. The eIDSR system is used to track 28 priority notifiable diseases in all 1,300 health facilities in the country [29]. The platform was developed using the country’s existing District Health Information System. However, the 1-1-7 system is presently not integrated into eIDSR. Going forward, if the Government maintains the 1-1-7 system, it is critical to ensure its interoperability and integration with the eIDSR system to open the opportunity to connect case-based reporting of notifiable diseases with event-based mortality surveillance to rapidly detect outbreaks and initiate public health response.

The expectation that the 1-1-7 system was going to be successfully converted to a routine mortality surveillance system does not seem to have been fulfilled given that the system only captured about 12% of the expected deaths in the aftermath of Ebola. The low level of death reporting to the 1-1-7 system after the epidemic ended was likely due to several factors including the lack of continued social mobilization to promote reporting and the lack of a clearly communicated government policy for routine use of the system. Sierra Leone’s implementation of the 1-1-7 system holds important lessons for the development and sustainability of similar telephone-based surveillance systems in sub-Saharan Africa. As reminded by the 2018–2020 Ebola outbreaks in the Democratic Republic of Congo and the ongoing COVID-19 pandemic, surveillance tools such as the 1-1-7 system when coupled with adequate public engagement can facilitate early detection of cases and deaths to curb disease spread.

### Limitations

Our assessment has limitations. Duplicate reporting may have been more frequent in the early stages of the epidemic in 2014 when the capacity to respond to deaths was at times unable to meet the demand for safe burial services. Improvements in response capacity in 2015 may have reduced the likelihood of families placing repeated calls for the same death in trying to ensure a safe burial. The number of duplicate reports likely stabilized starting in 2015 when response capacity improved. Duplicate records only accounted for about 5% of the total records in the database of deaths reported between December 2016 and April 2017. Assuming duplicate reporting level was similar between 2015 and 2019, it is unlikely that duplicate reporting substantially influenced the overall reporting trends. The descriptive trends provided in the paper are meant to help lay a foundation to examine and discuss the reporting motivations in a post-Ebola-outbreak context. Additional research could consider leveraging the recently launched Sierra Leone Ebola Database [30], which contains deduplicated, anonymized, linked data on 1-1-7 alerts, Ebola cases, laboratory results, Ebola Treatment Unit, Ebola Treatment Unit clinical records, and burial records, to ascertain death reporting trends using case-based data that account for duplicates.

The generalizability of the results of our telephone survey with death reporters also have limitations. For instance, the sample of respondents were mostly men. We do not know if this was because proportionally more men reported deaths to the 1-1-7 call center or whether it was due to a systematic bias of higher unsuccessful call rates to women who reported deaths. Lastly, our assessment only targeted individuals who reported deaths to the 1-1-7 system in order to understand their motivations for reporting. People who had deaths in their households but failed to report may be demographically different from our sample and held concerns that prohibited them from reporting. Future research should consider using qualitative approaches to better understand barriers to death reporting among household heads who fail to report deaths in their households.

### Conclusions

Support for compliance with government death reporting policy motivated users of the 1-1-7 system in Sierra Leone after the Ebola epidemic ended. Increased motivation to report deaths that resembled Ebola in the post-outbreak setting may have been influenced by knowledge and experiences from the prolonged epidemic. Post-Ebola-outbreak periods offer an opportunity for instituting routine mortality reporting, as people have been sensitized about the importance of reporting through the experiences of the outbreak. Transitioning the system to a routine mortality surveillance tool may require a robust social mobilization component [31] to match the high reporting levels during the outbreak, which exceeded more than 100% of the expected deaths in 2015. As global health security efforts try to strengthen surveillance systems [32], routine use of death reporting systems like 1-1-7 could play an important role in early detection of clusters of deaths linked to potential infectious disease outbreaks including Ebola.

## References

[1] World Health Organization. Ebola situation report—30 March 2016 2016 [cited 2018 March 19]. Available from: http://apps.who.int/iris/bitstream/10665/204714/1/ebolasitrep_30mar2016_eng.pdf.

[2] Richards P, Amara J, Ferme MC, Kamara P, Mokuwa E, Sheriff AI, et al Social pathways for Ebola virus disease in rural Sierra Leone, and some implications for containment. PLoS neglected tropical diseases. 2015;9(4):e0003567 Epub 2015/04/18. 10.1371/journal.pntd.0003567 25886400 PMC4401769

[3] Tiffany A, Dalziel BD, Kagume Njenge H, Johnson G, Nugba Ballah R, James D, et al Estimating the number of secondary Ebola cases resulting from an unsafe burial and risk factors for transmission during the West Africa Ebola epidemic. PLoS neglected tropical diseases. 2017;11(6):e0005491 Epub 2017/06/24. 10.1371/journal.pntd.0005491 28640823 PMC5480832

[4] Curran KG, Gibson JJ, Marke D, Caulker V, Bomeh J, Redd JT, et al Cluster of Ebola Virus Disease Linked to a Single Funeral—Moyamba District, Sierra Leone, 2014. MMWR Morb Mortal Wkly Rep. 2016;65(8):202–5. Epub 2016/03/05. 10.15585/mmwr.mm6508a2 . 26938950

[5] Chowell G, Nishiura H. Transmission dynamics and control of Ebola virus disease (EVD): a review. BMC Med. 2014;12:196 Epub 2014/10/11. 10.1186/s12916-014-0196-0 25300956 PMC4207625

[6] Government of Sierra Leone. National communication strategy for Ebola response in Sierra Leone 2014 [cited 2018 March 1]. Available from: http://ebolacommunicationnetwork.org/wp-content/uploads/2014/10/National-Ebola-Communication-Strategy_FINAL.pdf.

[7] Government of Sierra Leone. Ebola big idea—a NERC coordinated national media campaign 2015 [cited 2018 March 1]. Available from: http://nerc.sl/sites/default/files/docs/Ebola%20Big%20Idea.pdf.

[8] Laverack G, Manoncourt E. Key experiences of community engagement and social mobilization in the Ebola response. Glob Health Promot. 2016;23(1):79–82. 10.1177/1757975915606674 . 26518037

[9] Fast SM, Mekaru S, Brownstein JS, Postlethwaite TA, Markuzon N. The Role of Social Mobilization in Controlling Ebola Virus in Lofa County, Liberia. PLoS Curr. 2015;7 10.1371/currents.outbreaks.c3576278c66b22ab54a25e122fcdbec1 26075140 PMC4455978

[10] Funk S, Ciglenecki I, Tiffany A, Gignoux E, Camacho A, Eggo RM, et al The impact of control strategies and behavioural changes on the elimination of Ebola from Lofa County, Liberia. Philos Trans R Soc Lond B Biol Sci. 2017;372(1721). 10.1098/rstb.2016.0302 28396473 PMC5394640

[11] Jalloh MF, Sengeh P, Monasch R, Jalloh MB, DeLuca N, Dyson M, et al National survey of Ebola-related knowledge, attitudes and practices before the outbreak peak in Sierra Leone: August 2014. BMJ Glob Health. 2017;2(4):e000285 10.1136/bmjgh-2017-000285 29259820 PMC5728302

[12] Lawrence P, Danet N, Reynard O, Volchkova V, Volchkov V. Human transmission of Ebola virus. Curr Opin Virol. 2017;22:51–8. 10.1016/j.coviro.2016.11.013 . 28012412

[13] Miller LA, Stanger E, Senesi RG, DeLuca N, Dietz P, Hausman L, et al Use of a nationwide call center for Ebola response and monitoring during a 3-day house-to-house campaign—Sierra Leone, September 2014. MMWR Morb Mortal Wkly Rep. 2015;64(1):28–9. Epub 2015/01/16. 25590683 PMC4584796

[14] Alpren C, Jalloh MF, Kaiser R, Diop M, Kargbo S, Castle E, et al The 117 call alert system in Sierra Leone: from rapid Ebola notification to routine death reporting. BMJ Glob Health. 2017;2(3):e000392 10.1136/bmjgh-2017-000392 28948044 PMC5595198

[15] Nielsen CF, Kidd S, Sillah AR, Davis E, Mermin J, Kilmarx PH. Improving burial practices and cemetery management during an Ebola virus disease epidemic—Sierra Leone, 2014. MMWR Morb Mortal Wkly Rep. 2015;64(1):20–7. Epub 2015/01/16. 25590682 PMC4584795

[16] Lee-Kwan SH, DeLuca N, Bunnell R, Clayton HB, Turay AS, Mansaray Y. Facilitators and Barriers to Community Acceptance of Safe, Dignified Medical Burials in the Context of an Ebola Epidemic, Sierra Leone, 2014. J Health Commun. 2017;22(sup1):24–30. Epub 2017/08/31. 10.1080/10810730.2016.1209601 . 28854130

[17] CAFOD. Keeping the faith: the role of faith leaders in the Ebola response. Freetown: CAFOD Christian AID, Tear Fund, Islamic Relief Worldwide, 2015.

[18] Bedrosian SR, Young CE, Smith LA, Cox JD, Manning C, Pechta L, et al Lessons of Risk Communication and Health Promotion—West Africa and United States. MMWR Suppl. 2016;65(3):68–74. 10.15585/mmwr.su6503a10 . 27386834

[19] Gillespie AM, Obregon R, El Asawi R, Richey C, Manoncourt E, Joshi K, et al Social Mobilization and Community Engagement Central to the Ebola Response in West Africa: Lessons for Future Public Health Emergencies. Glob Health Sci Pract. 2016;4(4):626–46. 10.9745/GHSP-D-16-00226 28031301 PMC5199179

[20] eHealthAfrica. 117 and district call log analysis monthly reports. Freetown: eHealth Africa, 2019.

[21] Vandenbroucke JP, von Elm E, Altman DG, Gotzsche PC, Mulrow CD, Pocock SJ, et al Strengthening the Reporting of Observational Studies in Epidemiology (STROBE): explanation and elaboration. International journal of surgery (London, England). 2014;12(12):1500–24. Epub 2014/07/22. 10.1016/j.ijsu.2014.07.014 . 25046751

[22] OpenDataKit. ODK: Tools for the common case 2019 [cited 2017 April 1]. Available from: https://opendatakit.org/software.

[23] Marston BJ, Dokubo EK, van Steelandt A, Martel L, Williams D, Hersey S, et al Ebola Response Impact on Public Health Programs, West Africa, 2014–2017. Emerg Infect Dis. 2017;23(13). Epub 2017/11/21. 10.3201/eid2313.170727 29155674 PMC5711323

[24] Jalloh MF, Li W, Bunnell RE, Ethier KA, O’Leary A, Hageman KM, et al Impact of Ebola experiences and risk perceptions on mental health in Sierra Leone, July 2015. BMJ Global Health. 2018;3(2). 10.1136/bmjgh-2017-000471 29607096 PMC5873549

[25] UnitedNations. Population dynamics 2019 [cited 2020 June 12]. Available from: https://population.un.org/wpp/DataQuery/.

[26] Nuriddin A, Jalloh MF, Meyer E, Bunnell R, Bio FA, Jalloh MB, et al Trust, fear, stigma and disruptions: community perceptions and experiences during periods of low but ongoing transmission of Ebola virus disease in Sierra Leone, 2015. BMJ Glob Health. 2018;3(2):e000410 Epub 2018/04/10. 10.1136/bmjgh-2017-000410 29629189 PMC5884263

[27] Frieden TR, Damon IK. Ebola in West Africa—CDC's Role in Epidemic Detection, Control, and Prevention. Emerg Infect Dis. 2015;21(11):1897–905. 10.3201/eid2111.150949 26484940 PMC4622264

[28] WHO. Sierra Leone leads the way in Africa with fully functional electronic disease surveillance system 2019 [cited 2020 February 12]. Available from: https://www.afro.who.int/news/sierra-leone-leads-way-africa-fully-functional-electronic-disease-surveillance-system.

[29] U.S. Centers for Disease Control and Prevention. Ebola Outbreak Sparks Disease Surveillance Transformation in Sierra Leone 2019 [cited 2020 June 6]. Available from: https://www.cdc.gov/globalhealth/healthprotection/fieldupdates/fall-2019/sierra-leone-surveillance.html.

[30] U.S. Centers for Disease Control and Prevention. Data Hosting: Sierra Leone Ebola Database (SLED) 2020 [cited 2020 June 6]. Available from: https://www.cdc.gov/rdc/b1datatype/sled.htm.

[31] Pedi D, Gillespie A, Bedson J, Jalloh MF, Jalloh MB, Kamara A, et al The Development of Standard Operating Procedures for Social Mobilization and Community Engagement in Sierra Leone During the West Africa Ebola Outbreak of 2014–2015. J Health Commun. 2017;22(sup1):39–50. 10.1080/10810730.2016.1212130 . 28854137

[32] Heymann DL, Chen L, Takemi K, Fidler DP, Tappero JW, Thomas MJ, et al Global health security: the wider lessons from the west African Ebola virus disease epidemic. Lancet. 2015;385(9980):1884–901. Epub 2015/05/20. 10.1016/S0140-6736(15)60858-3 25987157 PMC5856330

